# Air concentrations of volatile compounds near oil and gas production: a community-based exploratory study

**DOI:** 10.1186/1476-069X-13-82

**Published:** 2014-10-30

**Authors:** Gregg P Macey, Ruth Breech, Mark Chernaik, Caroline Cox, Denny Larson, Deb Thomas, David O Carpenter

**Affiliations:** Center for Health, Science, and Public Policy, Brooklyn Law School, Brooklyn, New York USA; Global Community Monitor, Richmond, California USA; Environmental Law Alliance Worldwide, Eugene, Oregon USA; Center for Environmental Health, Oakland, California USA; Powder River Basin Resource Council, Clark, Wyoming USA; Institute for Health and the Environment, University at Albany, Rensselaer, New York USA

**Keywords:** Benzene, Community monitoring, Formaldehyde, Grab and passive samples, Hydraulic fracturing, Hydrogen sulfide, Oil and gas

## Abstract

**Background:**

Horizontal drilling, hydraulic fracturing, and other drilling and well stimulation technologies are now used widely in the United States and increasingly in other countries. They enable increases in oil and gas production, but there has been inadequate attention to human health impacts. Air quality near oil and gas operations is an underexplored human health concern for five reasons: (1) prior focus on threats to water quality; (2) an evolving understanding of contributions of certain oil and gas production processes to air quality; (3) limited state air quality monitoring networks; (4) significant variability in air emissions and concentrations; and (5) air quality research that misses impacts important to residents. Preliminary research suggests that volatile compounds, including hazardous air pollutants, are of potential concern. This study differs from prior research in its use of a community-based process to identify sampling locations. Through this approach, we determine concentrations of volatile compounds in air near operations that reflect community concerns and point to the need for more fine-grained and frequent monitoring at points along the production life cycle.

**Methods:**

Grab and passive air samples were collected by trained volunteers at locations identified through systematic observation of industrial operations and air impacts over the course of resident daily routines. A total of 75 volatile organics were measured using EPA Method TO-15 or TO-3 by gas chromatography/mass spectrometry. Formaldehyde levels were determined using UMEx 100 Passive Samplers.

**Results:**

Levels of eight volatile chemicals exceeded federal guidelines under several operational circumstances. Benzene, formaldehyde, and hydrogen sulfide were the most common compounds to exceed acute and other health-based risk levels.

**Conclusions:**

Air concentrations of potentially dangerous compounds and chemical mixtures are frequently present near oil and gas production sites. Community-based research can provide an important supplement to state air quality monitoring programs.

**Electronic supplementary material:**

The online version of this article (doi:10.1186/1476-069X-13-82) contains supplementary material, which is available to authorized users.

## Background

New drilling and well stimulation technologies have led to dramatic shifts in the energy market. The Energy Information Administration forecasts that by the 2030s, the United States will become a net exporter of petroleum liquids such as shale oil [1]. Already an exporter of natural gas, the U.S. will retrieve nearly half of its gas from shale formations by that time [2]. Reserves such as shale oil and gas are referred to as “unconventional” because fuels within them do not readily flow to the surface [3]. Instead, they are distributed among tight sandstone, shale, and other geologic strata. Intensive practices are used to retrieve them, such as directional drilling (many kilometres underground and one or more kilometres horizontally through a formation) and hydraulic fracturing to break up the formation and ensure movement through source rock (using millions of gallons of water mixed with chemicals and sand, or “proppants”) [4]. These technologies present public health challenges, including threats to air quality [5–7].

Unconventional oil and gas (hereinafter “UOG”) development and production involve multiple sources of physical stressors (e.g., noise, light, and vibrations) [6], toxicants (e.g., benzene, constituents in drilling and hydraulic fracturing fluids) [8], and radiological materials (e.g., technologically-enhanced, naturally-occurring radioactive material) [9], including air emissions [10, 11]. Air quality near UOG sites is an underexplored human health concern for several reasons. For a time, environmental scientists and regulators were primarily interested in potential impacts to surface and groundwater quality. High-profile impacts and the subsurface nature of technologies (e.g., hydraulic fracturing) encouraged this research trajectory [12]. This was true despite the fact that UOG development brings to the surface, in the case of natural gas, methane (78.3%), non-methane hydrocarbons (17.8%), nitrogen (1.8%), carbon dioxide (1.5%), and hydrogen sulfide (0.5%) [13]. These constituents, as well as emissions from combustion processes at the surface, are released to the air throughout the life cycle of a productive well [14].

Air emissions from UOG operations have been generally understood for some time – volatile organic compounds (VOCs), polycyclic aromatic hydrocarbons (PAHs), and criteria air pollutants such as NOx and PM_2.5_ can be released at the wellhead, in controlled burns (flaring), from produced water storage pits and tanks, and by diesel-powered equipment and trucks, among other sources [15]. Yet the full range of emissions from drilling, well completion, and other activities remains elusive. New source categories are discovered, emissions from life cycle stages such as transmission and well abandonment have yet to be determined, and even stages such as drilling continue to present uncertainty [16]. We do not understand the extent of drilling-related air emissions as pockets of methane, propane, and other constituents in the subsurface are disturbed and released to the atmosphere [17]. Emissions measurements during flowback vary by orders of magnitude [18]. These and other data gaps limit the accuracy of state and federal emissions inventories, which compile and track known emissions sources. Inventories are also limited by self-reporting and data collection, and rely in some cases on outmoded emissions factors [15]. Flawed inventories constrain human health risk assessment and other research [7] and slow the identification of phenomena such as photochemical ozone production during winter months [19].

State pollution monitoring networks also constrain research on the air impacts of UOG development. Historically, air quality monitoring targeted urban areas, and criteria air pollutants such as particulate matter and ozone precursors were the primary chemicals of concern [10]. Monitoring stations were designed to ensure compliance with National Ambient Air Quality Standards (NAAQS) for a half-dozen pollutants. Even networks that focus on oil and gas emissions, such as one operated by public health officials in Garfield County, Colorado, do not target individual well pads. The Garfield County network encompasses five sites to monitor a suite of VOCs and (at three sites) particulate matter, in a jurisdiction that covers nearly 3,000 square miles of complex terrain [20]. The Texas Commission on Environmental Quality has arguably the most extensive monitoring network for UOG air emissions in oil and gas regions. Its monitors were sited to minimize urban source impacts and target locations where the public might be exposed to air emissions [21]. Still, its networks can be sparse; there are five permanent monitoring stations in the Eagle Ford Shale region, where 7,000 oil and gas wells have been drilled since 2008 [22]. These and other limited networks potentially mask local hot spots, the effects of unique topography, and fugitive emissions at certain well pads.

Even a denser monitoring network taking continuous samples may be unable to capture the full range of air impacts of UOG operations. Sources of variability of air emissions and concentrations of VOCs and other pollutants near UOG sites include: (1) the spatial variability of UOG operations; (2) the discontinuous use of equipment such as diesel trucks, glycol dehydrators, separators, and compressors during preparation, drilling, hydraulic fracturing, well completion, and other stages; (3) the composition of shale and other formations and the specific constituents of the drilling and hydraulic fracturing fluids used on-site (which can influence the makeup of produced or flowback water stored in pits and tanks); (4) intermittent emissions from venting, flaring, and leaks; (5) the shifting location, spacing, and intensity of well pads in response to market conditions, improvements in technology, and regulatory changes; (6) the effects of wind, complex terrain, and microclimates; and (7) considerable differences among states in permitting, leak detection and repair, and other requirements [10, 16, 23–25]. Wind, for example, can influence outdoor and indoor concentrations of air pollutants. Brown et al. found that local air movement and mixing depth contribute to peak exposure to VOCs one mile from a compressor station [25]. Colborn et al. noted the role of wind and topography in higher VOC concentrations during winter months, when inversions trap air near ground level [10]. Fuller et al. identified wind speed and wind direction as significant predictors of indoor particulate matter levels near highways [26]. Similar variation can be found within and across geologic formations. Unconventional wells in the Barnett Shale play, for example, differ considerably in terms of reservoir quality, production rates, and recoverable gas [27]. Domestic shale gas plays exhibit even greater diversity, including depth and thickness of recoverable resources, the amount and range of chemicals present in produced water, and the presence of constituents such as bromide, naturally occurring radioactive material, hydrogen sulfide, and other toxic elements [23, 28].

These and other sources of variability, and the adaptive drilling and well completion techniques they encourage, complicate the design of setback and well spacing rules that are protective of the public. They also explain why air quality studies carried out in UOG regions yield conflicting results. For example, McKenzie et al. [11] found greater cumulative cancer risks and higher non-cancer hazard indices for residents living less than 0.5 miles from certain well pads in Colorado, while Bunch et al. [21] analyzed data from monitors focused on regional atmospheric concentrations in the Barnett Shale region and found no exceedance of health-based comparison values. Colborn et al. [10] gathered weekly, 24-hour samples 0.7 miles from a well pad in Garfield County, and noted a “great deal of variability across sampling dates in the numbers and concentrations of chemicals detected.” Eapi et al. [29] found substantial variation in fenceline concentrations of methane and hydrogen sulfide, which could not be explained by production volume, number of wells, or condensate volume at natural gas development sites.

Institutional factors also influence research on ambient air quality near UOG sites. Congressional exemption of oil and gas operations from provisions of the Clean Air Act, Clean Water Act, Safe Drinking Water Act, Emergency Planning and Community Right-to-Know Act, and other statutes limits data collection on the impacts of oil and gas development [30, 31]. In addition, the peer-reviewed literature is divided between “top-down” and “bottom-up” treatments of air quality. The first set of studies explores the impact of UOG operations on regional air quality, with a concern for methane emissions and ozone precursors in regions such as the Green River Basin in Wyoming [32], the Uintah Basin in northeastern Utah [33], and the Denver-Julesburg Basin, home of the Wattenberg Field in northeastern Colorado [34]. These studies rely on airborne and tower measurements, and are at times supplemented by ground measurements such as mobile monitoring.

For example, Petron et al. [35] found a strong alkane signature downwind from the Denver-Julesburg Basin, based on samples taken at a 300-m tall tower (the National Oceanic and Atmospheric Administration Boulder Atmospheric Observatory) and a mobile monitoring unit. In the Uintah Basin, where winter ozone levels exceeded the NAAQS 68 times in 2010, Helmig et al. [36] carried out vertical profiling of ozone precursors at a tower at the northern edge of a gas field. They found levels of atmospheric alkanes during temperature inversion events in 2013 that were 200–300 times greater than regional background. These and other “top-down” studies are also used to estimate methane leakage, which is helpful in comparing the climate-forcing impact of UOG to the use of coal-fired power plants. Loss rate estimates for methane and other hydrocarbons vary considerably by study, from 17% [37] (Los Angeles Basin) to 8.9% [38] (Uintah Basin) (6.2-11.7%, 95% C.I.) to 4% [35] (Denver-Julesburg Basin) (2.3-7.7%, 95% C.I.). A number of studies share the finding that EPA underestimates methane leakage rates across the life cycle (their estimate was 1.65% in 2013) [16], but others, extrapolating from emissions factors and/or direct measurement, produce estimates as low as 0.42% [18]. None of these studies attempts to characterize air concentrations within residential or publicly-accessible areas near UOG operations.

Other studies follow a “bottom-up” approach to air quality, which is limited by access to well pads and other infrastructure, the availability of a power source for monitoring equipment, the stage of operation underway, scheduled or unscheduled flashing, flaring, and fugitive releases, or movement of truck traffic and equipment at or near a well pad during a given sampling period. Thus, bottom-up studies vary in terms of distance to site, sample frequency, and chemicals targeted. This helps explain the range of findings in the published literature. Nevertheless, existing research gives support to resident reports of acute and long-term health symptoms and other reductions in quality of life. Even as they offer conflicting evidence of the relative importance of one stage of production or another to air emissions [10, 11], or differ in their ultimate conclusion regarding the existence [10, 11, 14, 35, 36, 39] or lack [21, 40, 41] of human health threats from air emissions, they find VOC concentrations in ambient air considerable distances from well pads, including in residential areas and public spaces.

The research questions that guide existing studies create a final barrier to our ability to characterize air emissions in UOG regions. Top-down studies are motivated by questions such as identifying sources of regional nonattainment of ozone standards, or estimating methane and other hydrocarbon leakage rates from UOG operations. Bottom-up research gathers data from one or a limited number of well pads, chosen for reasons such as access or cooperation by owners and operators. The data are used to discuss general exposure conditions for an often-hypothetical community, or used to derive a risk factor. In either mode of study, resident exposure does not directly motivate the sampling protocol. Rather, it is considered obliquely in a study’s choice of sample location (e.g., a one that is “near a small community”), assumed in measurements of concentrations within a certain distance of UOG activity, or ignored. What are missing from these studies are protocols grounded in a community’s experience of air quality impacts of UOG operations.

Our multi-state air quality monitoring study uses a community-based, participatory research (CBPR) design to explore conditions near UOG operations [42]. Its sampling protocol is based not on access to a well pad, data needs conditioned by an existing averaging standard, or regional policy concerns. Rather, we partnered with residents in UOG regions to measure air quality under circumstances that, given local knowledge of operations (e.g., emissions from particular equipment or intermittent practices) gained through daily routines (e.g., regular observation of well pads) and use of public and private spaces nearby (e.g., livestock movement, farming) were viewed by community members as potential threats to human health. Existing studies often lack a data set suitable for statistical analysis. When such analyses are occasionally imposed on bottom-up data sets, they explain only a fraction of the variance in air quality outcomes. For example, the highest R^2^ values in a study of 66 sites, which, due to the study’s broad spatial range was limited to measurements of methane and hydrogen sulfide, were 0.26 (H_2_S concentration vs. condensate volume nearby) and 0.17 (H_2_S and number of wells nearby) [29]. CBPR studies, by comparison, are place-based – they begin with the experience of a population in order to identify environmental stressors and explore the heterogeneity of circumstances under which they arise [43, 44]. Rather than discount these circumstances for lack of statistical power, they can be used to define the scope of confirmatory studies, tailor air quality monitoring networks and studies, or suggest novel pollution control measures and best management practices.

## Methods

We explore air quality at a previously neglected scale: near a range of UOG development and production sites that are the focus of community concern. Residents conducted sampling in response to operational conditions, odor events, and a history of the onset of acute symptoms. Residents selected sampling sites after they completed a training program run by Global Community Monitor (GCM), an organization that has developed and modified community-based sampling protocols for more than twenty years. Sampling is designed to obtain accurate readings of public exposure near UOG development in the part-per-billion range [45]. Training sessions followed a written manual on proper sampling protocol and included instruction by experienced members of GCM in a classroom setting for five hours. In addition, samplers were trained in the field to properly demonstrate Quality Assurance/Quality Control (QA/QC) methods, such as use of data sheets and chain of custody records, sampling procedures including not taking samples in the presence of vehicle traffic or other sources of VOCs, and protocols for storage and delivery to an analytic laboratory [45]. Chain of Custody forms provided by the laboratory were explained and filled out in exercises in which each sampler participated. The trainings for community-based air sampling and related QA/QC measures were developed in conjunction with the Environmental Protection Agency under the federal Environmental Monitoring for Public Access and Community Tracking (EMPACT) program, and refined in cooperation with agencies including the Health Services Department of Contra Costa County, California and the Delaware Department of Natural Resources [46, 47]. Any sample that did not meet QA/QC criteria was not included in the final data set.

Community monitors gauged industrial activity using field log sheets (“pollution logs”) that allow each resident to record what they see, hear, feel, smell, and taste in areas downwind of industrial activity as they go about their daily routines. Each community monitor participated voluntarily in data collection for this study. They provided consent to use data gathered with questionnaires that they co-designed as well as grab and passive samplers. Residents documented activity including: (a) visible emissions drifting off-site; (b) odors that appear to derive from a site; (c) acute health symptoms that occur while in proximity to a site or during a specific industrial activity; (d) audible sounds of particular equipment in use within the boundaries of an operating well pad or related infrastructure; and (e) visible activity on-site, including the number and types of heavy trucks and tanks, vehicle traffic, workers present and job categories, and physical changes such as noise and vibrations near certain equipment. Similar to a neighborhood police watch, each resident determined locations that they would continue to observe and potentially return to for sampling.

**Figure 1:**
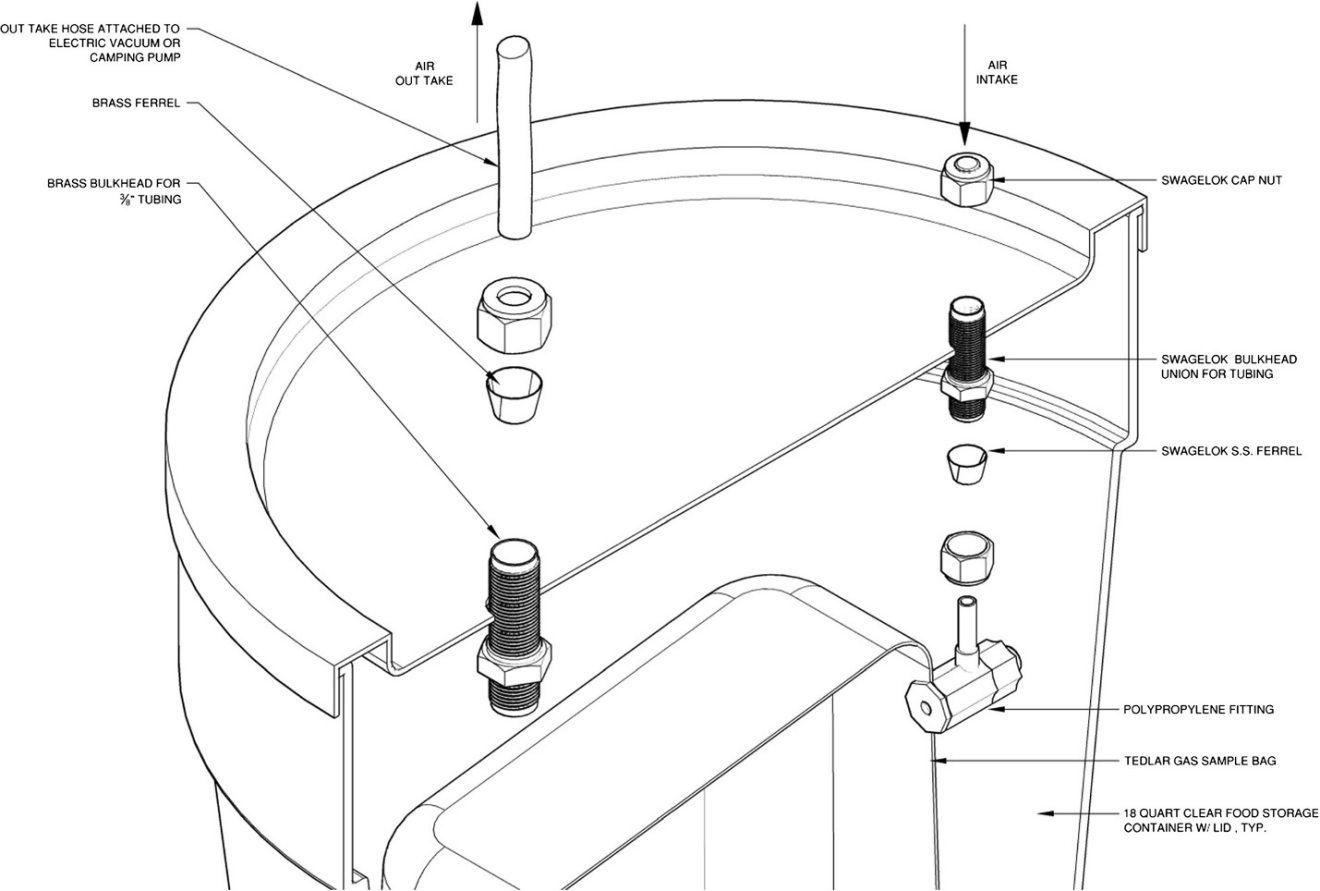
**Design of bucket grab sampling device.**

Sampling for volatile compounds other than formaldehyde was carried out using methods described in O’Rourke and Macey [48] and Larson et al. [49] using an evacuated sampling (“bucket”) vessel modelled after the Summa canister [50]. The bucket is inexpensive, portable, and consists of a 10-liter Tedlar bag and vacuum to take a grab sample of air for two to three minutes (Figure 1). Air is collected using a battery-operated pump that forces air out of the bucket. Negative pressure created inside the sealed bucket by the external vacuum pump opens the bag when a stainless steel bulkhead is opened. After taking the sample, the Tedlar bag is sealed and sent to an analytical laboratory. The bucket sampler operates on the same principle that Summa canisters employ. Rather than collect a sample in a stainless steel can, the bucket contains a special bag made of Tedlar to hold the sample. Bags are obtained from the laboratory that processes the sample and purged three times with pure nitrogen by the laboratory prior to use. GCM’s founder developed the sampling program under a project for Communities for a Better Environment, a non-profit organization founded in 1978 that provides legal, scientific, and technical assistance to heavily polluted communities. The device has been subjected to numerous validation tests organized by government agencies and independent laboratories [51–54]. Refinements include the use of field duplicates, which demonstrate no significant variation in results across comparison studies [45].

Residents collected 35 grab samples at locations of community concern, under conditions that would lead them to register a complaint with relevant authorities such as a county public health department or state oil and gas commission. Health symptoms contributed to the decision to take a grab sample on 29 occasions. The most common symptoms reported by samplers were headaches (17 reports), dizziness or light-headedness (13 reports), irritated, burning, or running nose (12 reports), nausea (11 reports), and sore or irritated throat (11 reports). Further details regarding each sample are provided in Additional file 1 (Tables S1 through S5).

In addition to grab samples, 41 formaldehyde badges were deployed in the five states targeting production facilities and compressor stations based on the results of pollution patrols. UMEx100 Passive Samplers for Formaldehyde are manufactured by SKC Inc. Samplers were placed near operating compressor stations and production facilities for a minimum of eight hours.

Samples were ultimately collected near production pads, compressor stations, condensate tank farms, gas processing stations, and wastewater and produced water impoundments in five states (Arkansas, Colorado, Ohio, Pennsylvania, and Wyoming). The states were chosen to reflect a diverse range of urban and rural communities, operations (e.g., number of wells permitted and developed), history of development, and stages of production (see Table 1).

**Table 1:** **Oil and gas operations by state**

State	Drilling permits issued (year)	Wells		Production		Setback requirements (dwellings and occupied structures)	Ambient air quality standards
		Drilled (year)	Producing (year)	Gas (Tcf) (year)	Oil (MMbbl) (year)		
AR	~ 890 (2012)^a^	--	8,538 (gas) (2012)^b^	1.15 (2012)^b^	6.59 (2012)^a^	200 ft. (from produced fluids storage tanks to habitable dwelling)	20 ppm (5 min.); 80 ppb (8-hr.) (H_2_S)^c^
	~ 1,090 (2011)^a^					300 ft. (from produced fluids storage tanks to school, hospital, or other public use building)	
CO	4,025 (2013)^a^	--	46,697 (2014)^d^	1.71 (2012)^b^	64.88 (2013)^a^	500 ft. (from well to home or building, absent waiver)	--^c,^ ^e^
	3,775 (2012)^a^					1,000 ft. (from well to high occupancy building, absent hearing and approval)	
OH	903 (2012)^a^	553 (2012)^a^	51,739 (2012)^a^	.084 (2012)^b^	4.97 (2012)^a^	150 ft. (occupied dwelling in urbanized area, absent consent)	--^c,^ ^e^
	690 (2011)^a^					150 ft. (occupied or public dwelling, non-urban area)	
						200 ft. (occupied dwelling w/in drilling unit due to mandatory pooling)	
PA	4,617 (2013)^a^	2,174 (2013)^a^	55,812 (2011)^f^	2.26 (2012)^b^	2.7 (2011)^a^	500 ft. (from well bore to building or water well)	0.1 ppm (1-hr.); 0.005 ppm (24-hr.) (H_2_S)^c, e^
	4,090 (2012)^a^						
WY	3,230 (Sept. 2013-Aug. 2014)^a^	--	37,301 (2012)^a^	2.23 (2012)^b^	57.5 (2012)^a^	350 ft. (from wellhead, pumping unit, pit, production tank, and/or production equipment to residence, school, or hospital)	40 μg/m^3^ (half-hr. ave., 2x w/in 5 days) (H_2_S)^c, e^

^a^State agency data.

^b^U.S. Energy Information Administration data.

^c^In addition to National Ambient Air Quality Standards for criteria air pollutants and federal emissions standards – new source performance standards (40 C.F.R. §§ 60.5360 - 60.5430) and national emission standards for hazardous air pollutants (40 C.F.R. §§ 63.760 - 63.777) – applicable to the oil and gas industry.

^d^Personal communication with state agency.

^e^In addition to state emissions standards (e.g., VOC emissions from glycol dehydrators; green completions; valve requirements for pneumatic devices). See, for example, Colorado Department of Public Health and Environment’s revised Air Quality Control Commission Regulation Numbers 3, 6, and 7 (adopted 23 February 2014).

^f^Earthworks data.

Air samples were analyzed for 75 volatile organic compounds (VOCs), including benzene, ethylbenzene, acrylonitrile, methylene chloride, toluene, hexane, heptane, and xylene by ALS Laboratories (Simi Valley, CA 93065) using EPA Method TO-15 or TO-3 (methane) by gas chromatograph/mass spectrometer interface to a whole air preconcentrator. Formaldehyde samples were analyzed using EPA Method TO-11A, modified for the sampling device by high performance liquid chromatography with UV detection. Samples were also analyzed for 20 sulfur compounds by ASTM D 5504–08 using a gas chromatograph equipped with a sulfur chemiluminescence detector. All compounds with the exception of hydrogen sulfide and carbonyl sulfide were quantitated against the initial calibration curve for methyl mercaptan. Chemicals of concern were compared to U.S. Agency for Toxic Substances and Disease Registry (ATSDR) minimal risk levels (MRLs) and EPA Integrated Risk Information System (IRIS) cancer risk levels. MRLs are estimates of daily human exposure that can occur without appreciable risk of human health effects. They are derived for acute (1–14 days), intermediate (15–364 days), or chronic (365 days or longer) periods of exposure. The laboratory is certified by ten state departments of health or environment, the American Industrial Hygiene Association, and the U.S. Department of Defense.

## Results

Table 1 shows the diverse range of operation, including number of wells permitted and developed and setbacks from housing and other occupied structures, in UOG regions where grab and passive air samples were collected through partnership with community-based organizations.

### Air contaminants

We identified unique chemical mixtures at each sample location (see Tables S1 through S5 in Additional file 1). In addition, we identified eight volatile compounds at concentrations that exceeded ATSDR minimal risk levels (MRLs) or EPA Integrated Risk Information System (IRIS) cancer risk levels (see Table 2). Although our samples represent a single point in time, we compared concentrations to acute as well as chronic risk levels as many of the activities that generate volatile compounds near UOG operations are long-duration (the life cycle of an unconventional natural gas well can span several decades) [16]. Residents chose sample locations where odors and symptoms were the “norm” for the area, not a one-time event. In addition, a growing body of research suggests that peak (e.g., 1-hr. maximum), rather than average exposure to air emissions may better capture certain risks to human health [55–57].

**Table 2:** **ATSDR minimal risk levels and EPA IRIS cancer risk levels for chemicals of concern (all data in μg/m** ^**3**^ **)**

Chemical	ATSDR MRLs			IRIS cancer risk levels		
	Acute	Intermediate	Chronic	1/1,000,000	1/100,000	1/10,000
Benzene	29	20	10	.45	4.5	45
1,3 butadiene				0.03	0.3	3
Ethylbenzene	21,700	8,680	260			
Formaldehyde	49	37	10	0.08	0.8	8
N-hexane			2,115			
Hydrogen sulfide	98	28				
Toluene	3,750		300			
Xylenes	8,680	2,604	217			

**Figure 2:**
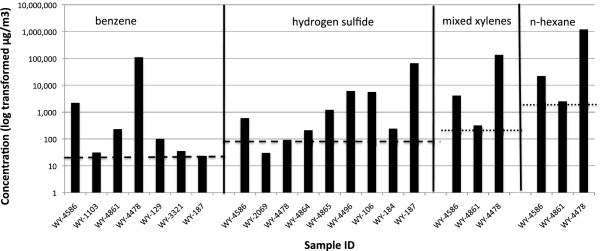
**Concentrations of volatile compounds exceeding health-based risk levels in samples collected in Wyoming.** Note log scale on y-axis. Dashed lines represent ATSDR intermediate-term MRLs. Dotted lines represent ATSDR chronic MRLs (not displayed: toluene, ethylbenzene, and formaldehyde).

**Figure 3:**
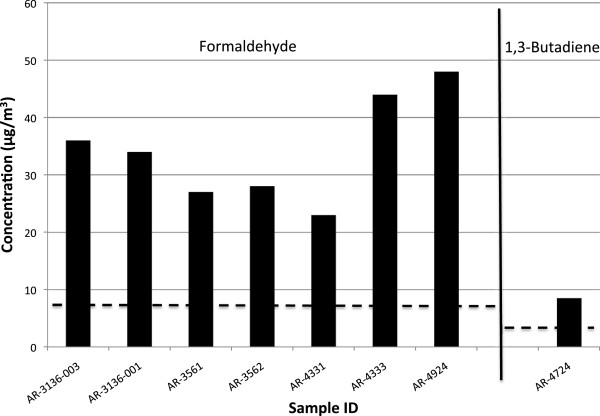
**Concentrations of volatile compounds exceeding health-based risk levels in samples collected in Arkansas.** Dashed lines represent EPA IRIS 1/10,000 cancer risk for formaldehyde and 1,3 butadiene.

**Figure 4:**
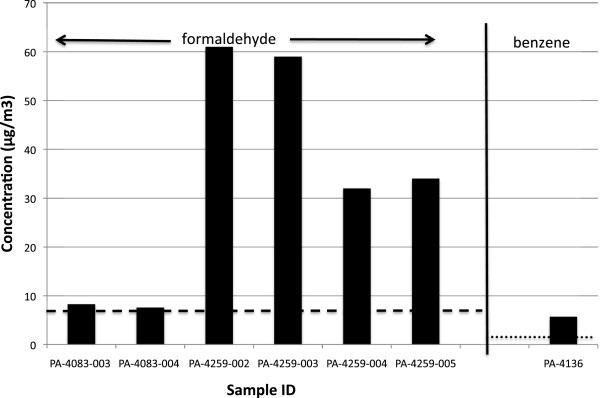
**Concentrations of volatile compounds exceeding health-based risk levels in samples collected in Pennsylvania.** Dashed line represents EPA IRIS 1/10,000 cancer risk for formaldehyde. Dotted line represents EPA IRIS 1/100,000 cancer risk for benzene.

Sixteen of the 35 grab samples, and 14 of the 41 passive samples, had concentrations of volatiles that exceeded ATSDR and/or EPA IRIS levels. ATSDR MRLs and EPA IRIS levels for chemicals of concern are provided in Table 2. The chemicals that most commonly exceeded these levels were hydrogen sulfide, formaldehyde, and benzene. Background levels for these chemicals are 0.15 μg/m^3^ for hydrogen sulfide, 0.25 μg/m^3^ for formaldehyde, and 0.15 μg/m^3^ for benzene [58–60]. Our samples that exceeded health-based risk levels were 90–66,000× background levels for hydrogen sulfide, 30-240× background levels for formaldehyde, and 35–770,000× background levels for benzene. Details of our results are presented in Tables 3, 4, and 5 and in Figures 2, 3, and 4 (greater detail is provided in Additional file 1). A state-by-state summary follows.

**Table 3:** **Concentrations of volatile compounds exceeding health-based risk levels in samples collected in Wyoming**

State/ID	County	Nearest infrastructure	Chemical	Concentration (μg/m ^3^)	ATSDR MRLs exceeded	EPA IRIS cancer risk exceeded
WY-4586	Fremont	5 m from separator	Hydrogen sulfide	590	I, A	n/a
WY-4586	Fremont	5 m from separator	Benzene	2,200	C, I, A	1/10,000
WY-4586	Fremont	5 m from separator	Toluene	1,400	C	n/a
WY-4586	Fremont	5 m from separator	Ethylbenzene	1,200	C	n/a
WY-4586	Fremont	5 m from separator	Mixed xylenes	4,100	C, I	n/a
WY-4586	Fremont	5 m from separator	n-hexane	22,000	C	n/a
WY-1103	Fremont	20 m from separator	benzene	31	C, I, A	1/100,000
WY-2069	Fremont	110 m from work-over rig^a^	Hydrogen sulfide	30	I	n/a
WY-4861	Fremont	5 m from separator	Benzene	230	C, I, A	1/10,000
WY-4861	Fremont	5 m from separator	Mixed xylenes	317	C	n/a
WY-4861	Fremont	5 m from separator	n-hexane	2,500	C	n/a
WY-4478	Park	25 m from separator	Hydrogen sulfide	91	I	n/a
WY-4478	Park	25 m from separator	Benzene	110,000	C, I, A	1/10,000
WY-4478	Park	25 m from separator	Toluene	270,000	C, A	n/a
WY-4478	Park	25 m from separator	Mixed xylenes	135,000	C, I, A	n/a
WY-4478	Park	25 m from separator	n-hexane	1,200,000	C	n/a
WY-129	Park	55 m from separator	benzene	100	C, I, A	1/10,000
WY-3321	Park	5 m from compressor	benzene	35	C, I, A	1/100,000
WY-4883-005	Park	5 m from compressor	Formaldehyde	46	C, I	1/10,000
WY-4864	Park	5 m from discharge canal	Hydrogen sulfide	210	I, A	n/a
WY-4865	Park	10 m from discharge canal	Hydrogen sulfide	1,200	I, A	n/a
WY-4496	Park	20 m from well pad	Hydrogen sulfide	6,100	I, A	n/a
WY-106	Park	Adjacent to discharge canal	Hydrogen sulfide	5,600	I, A	n/a
WY-184	Park	15 m from discharge canal	Hydrogen sulfide	240	I, A	n/a
WY-187	Park	15 m from discharge canal	Hydrogen sulfide	66,000	I, A	n/a
WY-187	Park	15 m from discharge canal	Benzene	23	C, I	1/100,000

C = chronic; A = acute; I = intermediate.

^a^Infrastructure used to pull and replace a well completion.

**Table 4:** **Concentrations of volatile compounds exceeding health-based risk levels in samples collected in Arkansas**

State/ID	County	Nearest infrastructure	Chemical	Concentration (μg/m ^3^)	ATSDR MRLs exceeded	EPA IRIS cancer risk exceeded
AR-3136-003	Faulkner	355 m from compressor	Formaldehyde	36	C	1/10,000
AR-3136-001	Cleburne	42 m from compressor	Formaldehyde	34	C	1/10,000
AR-3561	Cleburne	30 m from compressor	Formaldehyde	27	C	1/10,000
AR-3562	Faulkner	355 m from compressor	Formaldehyde	28	C	1/10,000
AR-4331	Faulkner	42 m from compressor	Formaldehyde	23	C	1/10,000
AR-4333	Faulkner	237 m from compressor	Formaldehyde	44	C, I	1/10,000
AR-4724	Van Buren	42 m from compressor	1,3-butadiene	8.5	n/a	1/10,000
AR-4924	Faulkner	254 m from compressor	Formaldehyde	48	C, I	1/10,000

C = chronic; I = intermediate.

**Table 5:** **Concentrations of volatile compounds exceeding health-based risk levels in samples collected in Pennsylvania**

State/ID	County	Nearest infrastructure	Chemical	Concentration (μg/m ^3^)	ATSDR MRLs exceeded	EPA IRIS cancer risk exceeded
PA-4083-003	Susquehanna	420 m from compressor	Formaldehyde	8.3		1/10,000
PA-4083-004	Susquehanna	370 m from compressor	Formaldehyde	7.6		1/100,000
PA-4136	Washington	270 m from PIG launch^a^	Benzene	5.7		1/100,000
PA-4259-002	Susquehanna	790 m from compressor	Formaldehyde	61	C, I, A	1/10,000
PA-4259-003	Susquehanna	420 m from compressor	Formaldehyde	59	C, I, A	1/10,000
PA-4259-004	Susquehanna	230 m from compressor	Formaldehyde	32	C	1/10,000
PA-4259-005	Susquehanna	460 m from compressor	Formaldehyde	34	C	1/10,000

C = chronic; A = acute; I = intermediate.

^a^Launching station for pipeline cleaning or inspection tool.

### Wyoming (Park County)

Nine of the ten grab samples contained volatiles above ATSDR MRLs or EPA IRIS risk levels. Seven contained high concentrations of hydrogen sulfide (one was over 600× the ATSDR acute MRL) and three contained high levels of benzene, including one over 12,000× the ATSDR acute MRL. The sample with the highest benzene concentrations also contained 480,000 micrograms per cubic meter of heptane, 3,100,000 micrograms per cubic meter of pentane, and 4,100,000 micrograms per cubic meter of butane, all hydrocarbons that are frequently associated with methane. These hydrocarbon concentrations exceeded occupational health standards (NIOSH recommended exposure limits). Four of the seven samples with high levels of hydrogen sulfide were taken in northeast Park County (near Deaver), and three of the four samples with high benzene levels were taken in northwest Park County (near Clark). One of the five passive samples contained formaldehyde at levels that exceeded ATSDR MRLs and the 1/10,000 cancer risk level (Table 3, Figure 2).

### Wyoming (Fremont County)

Four of the five grab samples contained volatiles at concentrations that exceeded ATSDR MRLs or EPA IRIS risk levels. One sample contained six volatiles exceeding these levels, including benzene at 75× the ATSDR acute MRL and 22× the EPA IRIS 1/10,000 cancer risk level. A second sample contained three volatiles exceeding ATSDR or EPA IRIS levels and also contained 4,167,000 micrograms per cubic meter of methane, an amount that exceeds its occupational health standard (Threshold Limit Value). None of the passive samples contained volatiles at concentrations that exceeded ATSDR MRLs or EPA IRIS cancer risk levels (Table 3, Figure 2).

### Arkansas (Cleburne, Faulkner, and Van Buren Counties)

One of the 8 grab samples, and 7 of the 13 passive samples, contained volatiles above ATSDR MRLs or EPA IRIS risk levels. One of the passive samples (taken at a residence) had formaldehyde levels that were close to the ATSDR MRL and exceeded EPA’s 1/10,000 cancer risk level (Table 4, Figure 3).

### Pennsylvania (Susquehanna County)

One of the four grab samples contained benzene at concentrations that exceeded the EPA 1/100,000 cancer risk level. Six of the ten passive samples contained formaldehyde at levels that exceeded ATSDR MRLs or EPA IRIS risk levels. Two of the samples exceeded both the acute MRL and the 1/10,000 cancer risk level (Table 5, Figure 4).

### Colorado (Boulder and Weld Counties)

One of the five grab samples contained 41 micrograms per cubic meter of hydrogen sulfide and exceeded the ATSDR intermediate MRL. None of the passive samples had volatiles exceeding the ATSDR MRLs or EPA IRIS risk levels.

### Ohio (Athens, Carroll, and Trumbull Counties)

None of the four grab samples or five passive samples contained volatiles at concentrations that exceeded the ATSDR MRLs or EPA IRIS risk levels.

### State air quality monitoring survey

We reviewed air quality monitoring by state agencies in the five states covered by our sampling. We reviewed one study in Arkansas, seven in Colorado, one in Ohio, four in Pennsylvania, and one in Wyoming. Most of the studies measured VOC levels, two included hydrogen sulfide, and seven included methane and/or other hydrocarbons. Sampling durations ranged from four hours to 24 months; five of the studies lasted more than four weeks. Target compounds were detected in all studies that have been completed, including mixtures of 42 non-methane VOCs. None of the studies concluded that detected compounds posed significant human health risk (Table 6).

**Table 6:** **Five-state survey of air quality monitoring studies, unconventional oil and gas operations**

Agency (year)	Target compound	Sampling equipment	Sample sites	Duration	Representative findings
ADEQ (2011)	VOCs (total) NO NO_2_	PID (fixed) PID (handheld)	4 compressor stations 6 drilling sites 3 well sites (fracking) 1 upwind	1 d (4–6 hrs.)	VOCs “almost always below or near detection limits” VOCs at drilling sites elevated (ave. 38–678 ppb; max. 350–5,321 ppb) NO/NO_2_ rarely exceed detection limits
CDPHE (2012)	NMOCs (78) Methane	Canister	1 well pad (Erie)	3 wks.	Detects = 42 of 78 compounds in >75% of samples Benzene “well within EPA’s acceptable cancer risk range” Acute and chronic HQs “well below” 1
CDPHE (2009)	NMOCs (78) VOCs PM_2.5_	Canister PID (handheld) Filter (handheld)	8 wells (4 drilling, 4 completion)	1 d	Total NMOC ave. 273 – 8,761 ppb at 8 sites Total VOC ave. 6–3,023 ppb at 8 sites PM_2.5_ ave. 7.3 - 16.7 μg/m^3^ at 8 sites
CDPHE, GCPHD (2007)	VOCs (43) PM_10_	Canister Filter	14 sites 7 sites	24 mos.	Detects = 15 of 43 compounds Benzene ave. 28.2 μg/m^3^, max 180 μg/m^3^ (grab) Toluene ave. 91.4 μg/m^3^, max 540 μg/m^3^ (grab)
CDPHE (2003–2012)	NMOCs Carbonyls	Canister	5 sites (2003) 6 sites (2006) 3+ sites (2012)	2 mos.	Methane ave. 2,535 ppb (Platteville) vs. (1,780 ppb Denver) Top NMOCs in Platteville = ethane, propane, butane Benzene, toluene higher in Platteville
CDPHE (2002)	VOCs (42) SO_2_ NO, NO_2_	Canister Continuous	2 well sites 1 residential 1 active flare 2 up-, down-valley 1 background	1 mo.	Detects = 6 of 42 VOCs Benzene in 6 of 20 (2.2-6.5 μg/m^3^) Toluene in 18 of 20 (1.5-17 μg/m^3^)
OEPA (2014)	VOCs (69) VOCs PM_10_/PM_2.5_ H_2_S CO	Canister GC/MS Filter	1 well site 1 remote site	12 mos.	Ongoing; data update provided in February 2014 Detects include BTEX, alkanes (e.g., ethane, hexane), H_2_S Second site planned near processing plant
PA DEP (2010)	VOCs (48) Alkanes Leak detection	Canister OP-FTIR GC/MS FLIR	2 compressor stations 1 condensate tank 1 wastewater impoundment 1 background	5 wks.	Detects include methane, ethane, propane, benzene (max. 758 ppb) No conc.’s “that would likely trigger air-related health issues” Fugitive gas stream emissions
PA DEP (2011)	VOCs (48) Alkanes Leak detection	Canister OP-FTIR GC/MS FLIR	2 compressor stations 1 completed well 1 well site (fracking) 1 well (tanks, separator) 1 background	4 wks.	Detects include BTEX (benzene max. 400 ppb), methylbenzenes No conc.’s “that would likely trigger air-related health issues” Fugitive emissions from condensate tanks, piping
PA DEP (2011)	VOCs (48) Alkanes	Canister OP-FTIR GC/MS	2 compressor stations 1 well site (flaring) 1 well site (drilling) 1 background	4 wks.	Detects include benzene (max. 400 ppb), toluene, ethylbenzene Natural gas constituent detects near compressor stations Conc.’s “do not indicate a potential for major air-related health issues”
PA DEP (2012)	Criteria VOCs/HAPs Methane H_2_S	“Full suite”	1 gas processing 2 large compressor stations 1 background	12 mos.	Ongoing; report due in 2014
WDEQ (2013)	VOCs/NMHCs Ozone Methane NO, NO_2_ PM_10_/PM_2.5_	Canister UV Photometric FID Chemiluminescence Beta Attenuation	7 permanent stations (e.g., Boulder, Juel Spring, Moxa) 3 mesonet stations (Mesa, Paradise Warbonnet) 2 mobile trailer locations (Big Piney, Jonah Field)	Ongoing	WDEQ mobile monitors placed at locations w/ oil & gas development Mini-SODAR also placed adjacent to Boulder permanent station “Relatively low concentrations” of VOCs found in canister samples VOCs “consistently higher” at Paradise site (near oil & gas sources)

BTEX = benzene, toluene, ethylbenzene, and xylenes; FID = flame ionization detector; FLIR = forward looking infrared; GC/MS = gas chromatography/mass spectrometry; HAP = hazardous air pollutant; NAAQS = National Ambient Air Quality Standard; NMHC = non-methane hydrocarbon; NMOC = non-methane organic compound; OP-FTIR = open-path Fourier transform infrared; PID = photoionization detector; VOC = volatile organic compound.

## Discussion

We identified significant concentrations of four well-characterized chemicals: benzene, formaldehyde, hexane, and hydrogen sulfide. Benzene was detected at sample locations in Pennsylvania and Wyoming. Concentrations exceeded health-based risk levels by as many as several orders of magnitude. Previous studies similarly found benzene concentrations near oil and gas development [10, 11]. Our monitors detected benzene at higher concentrations (5.7 – 110,000 μg/m^3^) than those found in the published literature. The results are of concern given their proximity to subdivisions, homes, and farms. In Wyoming, multiple samples with high benzene concentrations were taken on residential property 30–350 yards from the nearest well, or on farmland along the perimeter of a well pad. Equipment included separators, compressor stations, discharge canals, and pipeline cleaning operations. The results suggest that existing regulatory setback distances from wells to residences may not be adequate to reduce human health risks [61]. Setbacks from wellheads to homes and other occupied structures cluster around the 150 to 500 feet range in the five states (see Table 1). We found high concentrations of volatile compounds at greater distances, including formaldehyde (up to 2,591 feet) and benzene (up to 885 feet). High levels of benzene near oil production wells indicate that EPA should revisit the extent to which oil wells are addressed in its new source performance standards [62].

Benzene is a known human carcinogen. Chronic exposure to benzene increases the risk of leukemia [63]. The increased risk occurs at low levels of exposure with no evidence of threshold level [64]. Benzene exposure increases risk of birth defects [65], including neural tube and other defects found near natural gas development [24]. Respiratory effects include pulmonary edema, acute granular tracheitis, laryngitis, and bronchitis [60].

UOG fields present multiple sources and exposure routes for benzene. Benzene occurs naturally in shale and other hydrocarbon deposits, and is vented, flared, or released as fugitive emissions along numerous points of production, such as wells, production tanks, compressors, and pipelines [6]. It can volatize and disperse from flowback and produced water at drilling sites and remain in the air for several days [66]. It was among the first pollutants found in air samples near shale gas operations [67]. Previous studies found benzene to be the largest contributor to excess lifetime cancer risk near gas fields [12]. Residents exposed to VOCs including benzene experience immediate health symptoms and illness. Within days after a flaring event at a Texas City refinery, children exhibited altered blood profiles, liver enzymes, and somatic symptoms [68]. Future research is needed to determine whether the concentrations of benzene we measured are due to continuous releases or flaring, fugitive emissions, or facility upsets.

Formaldehyde is another volatile compound that exceeded health-based risk levels near compressor stations in Arkansas, Pennsylvania, and Wyoming. As with benzene, there are known sources of formaldehyde emissions along the production chain. Formaldehyde is a product of incomplete combustion emitted by natural gas-fired reciprocating engines at compressor stations [69]. Formaldehyde is also formed from methane in the presence of sunlight, which may be an important source given significant amounts of methane that are known to escape from UOG sites [70]. But air monitoring studies, particularly in shale gas regions, either do not measure for formaldehyde [12, 14] or find it at lower concentrations. For example, the Barnett Shale Energy Education Council [71] found levels that did not pose a risk to human health. Colborn et al. [10] found formaldehyde and acetaldehyde in each of 46 samples with a mean of 1.0 part per billion by volume. In contrast, our CBPR framework resulted in the targeting of compressor stations for passive sampling, where diesel emissions likely account for the higher levels that we found. Our results are similar to the Fort Worth Natural Gas Air Quality Study, which found formaldehyde concentrations in areas with multiple large compressor engines [72]. We found high concentrations of formaldehyde near fourteen compressor stations in three states.

Formaldehyde is a suspected human carcinogen [73]. It can affect nearly every tissue in the human body, leading to acute (dermal allergies, asthma) and chronic (neuro-, reproductive, hematopoietic, genetic and pulmonary toxicity and cellular damage) health effects [74]. The science of childhood exposure to formaldehyde is progressing rapidly [75]. State agencies and international organizations continue to lower exposure limit values and guidelines for formaldehyde [76]. Our results exceed those guidelines. Symptoms reported by community members mirror the effects of acute formaldehyde exposure, which causes irritation of the eyes, nose, throat, and skin.

Other volatiles of concern included hexane and hydrogen sulfide. Hexane detects were most prevalent near oil and gas operations in Wyoming near well pads, compressor stations, separators, and produced water discharges. Other studies in oil and gas regions found hexane, but at low concentrations [10, 12]. The circumstances under which high concentrations of hexane were found in Wyoming suggest a combination of leaks, spills, and fugitive emissions as potential causes. Acute exposure to hexane affects the central nervous system, causing dizziness, nausea, and headache. Chronic effects include neurotoxicity [77].

We also found elevated levels of hydrogen sulfide in Wyoming along the chain of production (pump jacks, produced water discharge impoundments, discharge canals) and near a well pad in Colorado. Hydrogen sulfide is a broad-spectrum toxicant that can impact most organ systems [78]. As such, it contributes to a range of short- and long-term neurological, upper respiratory, and blood-related symptoms, including those that were prevalent among community samplers in Wyoming (headaches, dizziness, eye irritation, fatigue) [79]. Hydrogen sulfide is a natural component of crude oil and natural gas [5] and is released during many industrial processes. In addition, five samples from Wyoming exceeded ATSDR health-based risk levels for toluene and xylenes.

Health-based risk levels provide only a limited sense of potential human health impacts from air emissions. They do not fully account for vulnerable subpopulations, and toxicity values are available for a comparatively small number of compounds. The levels that we found for the above chemicals of concern suggest that state monitoring studies are incomplete. Recent state-funded projects found air volatiles at UOG sites that were either near detection limits or within acceptable limits to protect the public [80–82]. One area of agreement between our community-based and state monitoring studies concerns the presence of complex chemical mixtures. These mixtures demonstrate the contingent nature of ambient air quality near UOG infrastructure.

For example, one sample, taken midday in early winter near a well pad in Wyoming with clicking pneumatic pumps, found high concentrations of hydrogen sulfide, hexane, benzene, and xylenes. It also captured cyclohexane, heptane, octane, ethylbenzene, nonane, 1,2,4-trimethylbenzene, and 15 tentatively identified compounds (TICs). TICs are compounds that a device or analytic process is not designed to measure. Total VOC concentrations in the sample exceeded 1.6 million μg/m^3^, excluding methane. While toxicity values are not available for every TIC in our samples, they exceeded reference concentrations available for related compounds such as hexane [77]. Another sample taken in Arkansas, during autumn in the afternoon near a compressor station, captured 17 volatile compounds and five TICs. A third sample, near a separator shed in Wyoming in late autumn at midday, showed spikes in hydrogen sulfide, benzene, and hexane, 19 additional VOCs, and 15 TICs, with total VOC concentrations exceeding 25 million μg/m^3^, excluding methane. These and other complex mixtures are provided in Additional file 1.

The mixtures that we identified are related to sources commonly used in well pad preparation, drilling, well completion, and production, such as produced water tanks, glycol dehydrators, phase separators, compressors, pipelines, and diesel trucks [14]. They can be released during normal operating conditions and persist near ground level, especially in regions where topography encourages air inversions [83]. The toxicity of some constituents is well known, while others have little or no toxicity information available. Our findings of chemical mixtures are of clinical significance, even absent spikes in chemicals of concern. The chemical mixtures that we identified should be further investigated for their primary emissions sources as well as their potential cumulative and synergistic effects [84]. Clinical and subclinical effects of hydrocarbons such as benzene are increasingly found at low doses [85]. Chronic and subchronic exposure to chemical mixtures is of particular concern to vulnerable subpopulations, including children, pregnant women, and senior citizens [86].

Apart from chemicals of concern (including known and suspected human carcinogens) and chronic exposure to complex mixtures, our findings point to the value of community-based research to inform state testing protocols. Air quality near the diverse range of equipment and stages of UOG development is inherently complex. While states sometimes rely on state-of-the-art technologies such as wireless sensors to characterize local air quality, they continue to collect only a “snapshot” of near-field conditions. For example, Arkansas carried out a technologically ambitious program, placing multi-sensor gas monitors on five-foot tripods along each perimeter of a well pad at several sites. AreaRAEs (the trade name for a wireless monitor produced by RAE Systems) use electrochemical sensors to measure nitrous oxides and a photoionization detector to determine VOC concentration. The continuous monitors wirelessly transmitted data at five-second intervals over a four- to six-hour period (see Table 6). In addition, Arkansas Department of Environmental Quality (ADEQ) personnel carried handheld versions of the AreaRAE along the perimeter of the sites every one or two hours. While the study did not identify individual VOCs, it found that total VOC emissions at the edge of a well pad fluctuate wildly over a five-hour period. The agency concluded, “The spatial and temporal distribution of VOC concentrations at most drilling sites was significantly affected by monitor location, wind, and the interaction between location and wind direction” [81]. Other studies noted similar variation, although the extent to which short-term spikes and unique chemical mixtures might pose a risk to human health was not considered.

Community-based research can improve the spatial and temporal resolution of air quality data [87] while adhering to established methods. Our findings can inform and calibrate state monitoring and research programs. Additional file 1: Table S6 gives a more in-depth overview of community monitoring in action, including sample site selection factors, sources of public health concern at each site, and the range of infrastructure present and life cycle stage when samples were taken. For example, grab samples in Wyoming with some of the highest VOC concentrations were collected during production, as opposed to well completion (see Table S6, Additional file 1). The timing and location of our samples were driven by two primary factors: local knowledge gleaned from daily routines, and a history of chronic or subchronic symptoms reported by nearby residents. For example, a separator shed was targeted because of subchronic symptoms (dizziness, nausea, tight chest, nose and throat problems, metallic taste, and sweet smell) and loud sounds nearby (“hissing, clicking, and whooshing”). Well pads were selected based on impacts to livestock, pasture degradation from produced water, and observations of residents and farmers. Other samples were driven by observations of fugitive emissions, including vapor clouds, deposition, discoloration, and sounds (see Table S6 in Additional file 1).

Community-based research can identify mixtures, and their potential emissions sources, to prioritize for study of their additive, cumulative, and synergistic effects [88]. The mixtures can be used to determine source signatures [14] and isolate well pads for more intensive monitoring. Symptom-driven samples can define the proper length of a sampling period, which is often limited to days or weeks. They can inform equipment placement for continuous monitoring and facilitate a transition from exploratory to more purposive sampling. Testing informed by human health impacts, and more precise knowledge of the mix and spacing of sources that may contribute to them, contrasts with state efforts, which are limited by access to property, sources of electrical power, fixed monitoring sites, and the cooperation of well pad owners and operators. In these ways, community-based monitoring can extend the reach of limited public resources.

## Conclusions

Community-based monitoring near unconventional oil and gas operations demonstrates elevations in concentrations of hazardous air pollutants under a range of circumstances. Of special concern are high concentrations of benzene, hydrogen sulfide, and formaldehyde, as well as chemical mixtures linked to operations with observed impacts to resident quality of life.

## Electronic supplementary material

Additional file 1: **Contains six tables, including complete results from grab and passive sampling (Tables S1 through S5) and data on sample location selection in Wyoming (Table S6).** (DOC 174 KB)

## Abbreviations

ADEQ: Arkansas Department of Environmental Quality
ATSDR: Agency for Toxic Substances and Disease Registry
BTEX: benzene, toluene, ethylbenzene, and xylenes
CBPR: community-based participatory research
CDPHE: Colorado Department of Public Health and Environment
EMPACT: Environmental Monitoring for Public Access and Community Tracking
EPA: Environmental Protection Agency
FID: flame ionization detector
FLIR: forward looking infrared
GCM: Global Community Monitor
GC/MS: gas chromatography/mass spectrometry
GCPHD: Garfield County Public Health Department
HAP: hazardous air pollutant
IRIS: Integrated Risk Information System
MRL: minimal risk level
NAAQS: National Ambient Air Quality Standard
NIOSH: National Institute for Occupational Safety and Health
NMHC: non-methane hydrocarbon
NMOC: non-methane organic compound
OEPA: Ohio Environmental Protection Agency
OP-FTIR: open path Fourier transform infrared
PA DEP: Pennsylvania Department of Environmental Protection
PAH: polycyclic aromatic hydrocarbon
PID: photoionization detector
QA/QC: Quality Assurance/Quality Control
TIC: tentatively identified compound
UOG: unconventional oil and gas
VOC: volatile organic compound
WDEQ: Wyoming Department of Environmental Quality.
