# Supplementary material for: Air concentrations of volatile compounds near oil and gas production: a community-based exploratory study
Source: Environ Health. 2014 Oct 30;13:82. doi: 10.1186/1476-069X-13-82 (PMC4216869; doi:10.1186/1476-069X-13-82)
Supplement: Supplementary file 1 — Additional file 1: Contains six tables, including complete results from grab and passive sampling (Tables S1 through S5) and data on sample location selection in Wyoming (Table S6). (DOC 174 KB) [file 12940_2014_790_MOESM1_ESM.doc]

Table of Contents

Table S1. Arkansas Grab and Passive Sample Results and Symptoms by Location……………… 2

Table S2. Colorado Grab and Passive Sample Results and Symptoms by Location……………… 6

Table S3. Ohio Grab and Passive Sample Results and Symptoms by Location…………………... 7

Table S4. Pennsylvania Grab and Passive Sample Results and Symptoms by Location………….. 8

Table S5. Wyoming Grab and Passive Sample Results and Symptoms by Location……………... 10

Table S6. Grab Samples Collected on Pollution Patrol in Fremont and Park Counties, WY……... 18

**Table S1.** Arkansas Grab and Passive Sample Results and Symptoms by Location.

| **Location** | **Date (Sample ID)** | **Time** | **Type** | **Compound**  (μg/m^3^) | **TICs**  (μg/m^3^) | **Symptoms** |
| --- | --- | --- | --- | --- | --- | --- |
| 35.205301N 92.115837W | 11/4/13  (AR-4912) | 12:45 p.m. | Grab | Ethanol (79)  Acetone (80)  d-Limonene (8.7)  Methane (2.6 ppmV) | Hexamethyl-cyclotrisiloxane (28)  2,4-Dimethylheptane (56)  4-Methyloctane (37)  C12H26 Branched Alkane (23)  Dodecane (27) | **Smell**: Strong natural gas odorant smell mixed with sweet  **Feel**: Shaking hands |
| 35.213499N  92.02414W | 11/3/13  (AR-4913) | 12:55 p.m. | Grab | Ethanol (80)  Acetone (66)  Methane (3.9 ppmV) | 2,4-Dimethylheptane (25) | **Smell**: Natural gas odorant smell  **Feel**: Burning nostril |
| 35.213504N  92.02385W | 11/3/13  (AR-4198) | 11:55 p.m. | Grab | Ethanol (55)  Toluene (11)  Methane (4 ppmV) | 2,4-Dimethylheptane (23) | **See**: White smoke  **Smell**: Sewer, sweet, skunk, burnt chemical, natural gas odorant. Severe smell came and went in correlation with sounds from compressor  **Feel**: Headache, nauseous, sore throat  **Taste**: Sweet  **Hear**: Oscillating noise, rumble, running/idling |
| 35.213501N  92.02450W | 11/3/13  (AR-4914) | 11:45 p.m. | Grab | Ethanol (53)  Methane (2.5 ppmV) |  | **Smell**: Natural gas odorant smell  **Feel**: burning nose, stomach cramps, nausea |

| 35.28603N 92.282789W | 10/23/13  (AR-4724) | 3:23 p.m. | Grab | Propene (61)  Dichlorodifluoromethane (5.8)  1,3 Butadiene (8.5)  Ethanol (120)  Acetone (67)  THF (7.7)  n-Heptane (13)  4-Methyl-2-pentanone (5.7)  Toluene (56)  n-Octane (8.8)  Ethylbenzene (30)  m,p-Xylenes (45)  0-Xylene (14)  n-Nonane (15)  alpha-Pinene (62)  1,2,4-Trimethyl-benzene (5.2)  d-Limonene (83)  Methane  (7.4 ppmV) | C4H8 Alkene (35)  2-Butanol (28)  C10H16 Compound (22)  3-Carene (26)  Eucalyptol (29) | **See**: Chemical vapor  **Smell**: Antifreeze, sweet, skunk, burnt chemical, gasoline; smell was more intense on pad adjacent to station  **Feel**: Headache, nauseous, sore throat, itchy, skin became very irritated  **Taste**: Sweet  **Hear**: Oscillating noise, running/idling |
| --- | --- | --- | --- | --- | --- | --- |

| 35.221233N 92.121360W | 10/22/13  (AR-4701) | 1:43 p.m. | Grab | Ethanol (68)  Acetone (57)  Methane  (2.5 ppmV) |  | **See**: White dust  **Smell**: Chlorine, burnt chemical similar to when plastic burns  **Feel**: Headache, burning nose |
| --- | --- | --- | --- | --- | --- | --- |
| 35.165528N 92.254383W | 1/17/13  (AR-0215) | 10:00 a.m. | Grab | Ethyl Acetate (17) |  | **Smell**: Sewer, sweet, gas odorant  **Feel**: Headache, nauseous, sore throat, had fever for two days afterwards  **Taste**: Sweet, sour, battery acid |
| 35.205355N 92.12540W | 1/17/13  (AR-0214) | 8:05 a.m. | Grab | Ethyl Acetate (21) |  | **See**: Chemical vapor  **Smell**: Sweet, skunk, really strong, had to cover face  **Feel**: Headache, nauseous, burning eyes/nose/lips, dizzy, bloody nose, shaking, nose bleed, and tremors later that day  **Taste**: Sweet  **Hear**: Oscillating noise, running/idling |
| 35.205340N 92.12438W | 11/3/13  AR-4924-001) | 12:22 p.m. | Passive | Formaldehyde (48) |  | **See**: Chemical vapor  **Smell**: Sweet  **Feel**: Headache, coughing |
| 35.165510N 92.254621W | 11/3/13  (AR-4923-001) | 10:30 p.m. | Passive |  |  | **See**: Chemical vapor  **Hear**: Oscillating noise, rumble, running/idling |
| 35.121422N 92.213218W | 11/3/13  (AR-4915-001) | n/a | Passive |  |  |  |
| 35.165520N 92.254609W | 9/26/13  (AR-4333-001) | 3:05 p.m. | Passive | Formaldehyde (44) |  |  |
| 35.22328N 92.12572W | 9/26/13  (AR-4335-001) | 2:15 p.m. | Passive |  |  | **Smell**: Sweet, skunk, burnt chemical  **Feel**: Headache, nauseous, burning nose/throat, dizzy, itchy, concentration problem |
| 35.22308N 92.12588W | 9/26/13  (AR-4332-001) | 8:20 a.m. | Passive |  |  |  |
| 35.121422N 92.213218W | 9/25/13  (AR-4336-001) | 11:30 p.m. | Passive | Formaldehyde (51) |  |  |
| 35.205347N 92.12487W | 9/25/13  (AR-4331-001) | 4:30 p.m. | Passive | Formaldehyde (23) |  |  |
| 35.165516N 92.254615W | 9/25/13  (AR-4334-001) | 3:55 p.m. | Passive |  |  |  |
| 35.165522N 92.254602W | 8/12/13  (AR-3562-001) | 1:25 p.m. | Passive | Formaldehyde (28) |  |  |
| 35.22346N 92.12563W | 8/12/13  (AR-3561-001) | 12:50 p.m. | Passive | Formaldehyde (27) |  |  |
| 35.165563N 92.254621W | 7/18/13  (AR-3136-003) | 9:14 a.m. | Passive | Formaldehyde (36) |  |  |
| 35.28819N 92.284001W | 7/17/13  (AR-3136-002) | 1:04 p.m. | Passive |  |  |  |
| 35.165563N 92.254621W | 7/17/13  (AR-3136-001) | 11:37 a.m. | Passive | Formaldehyde (34) |  |  |

**Table S2.** Colorado Grab and Passive Sample Results and Symptoms by Location.

| **Location** | **Date**  **(Sample ID)** | **Time** | **Type** | **Compound**  (μg/m^3^) | **TICs**  (μg/m^3^) | **Symptoms** |
| --- | --- | --- | --- | --- | --- | --- |
| 40.00008N  105.067164W | 8/21/12  (CO-3460) | 10:25 p.m. | Grab | Toluene (7.1)  alpha-Pinene (9) | Unidentified Compound (27)  2-Ethyl-1-hexanol (75)  C12H26 Branched Alkane (25-75) |  |
| 40.2286N  105.22439W | 8/6/12  (CO-3190) | 6:00 a.m. | Grab | Ethanol (69) | Butane (22) |  |
| 40.3379N  105.34058W | 8/2/13  (CO-3402) | 6:15 p.m. | Grab | Ethanol (230)  Toluene (6.6)  Chlorobenzene (37)  d-Limonene (8.2)  Methane  (4.4 ppmV) | Propane (31) | **Smell**: Gas smell in waves  **Feel**: Nausea, lips tingle |
| 40.089891N  105.013863W | 7/31/12  (CO-3137) | 11:41 p.m. | Grab | Hydrogen Sulfide (41)  Toluene (5.5)  alpha-Pinene (26)  d-Limonene (8) | C8H16 Compound (29)  C11H24 Branched Alkane (37-78)  beta-Pinene (35)  2-Ethyl-1-hexanol (180)  C12H26 Branched Alkane (43-350)  Unidentified Compound (37)  Unidentified Compound (33)  Unidentified Compound (45)  Unidentified Compound (28) |  |
| 40.23342N  105.35470W | 7/30/12  (CO-3118) | 6:16 p.m. | Grab |  |  |  |
| 40.51626N  104.531097W | 10/28/13  (CO-4831-003) | 8:15 p.m. | Passive |  |  |  |
| 40.52282N  104.531100W | 10/28/13  (CO-4831-002) | 8:10 p.m. | Passive |  |  |  |
| 40.53135N  104.531211W | 10/28/13  (CO-4831-001) | 8:05 p.m. | Passive |  |  |  |

**Table S3.** Ohio Grab and Passive Sample Results and Symptoms by Location.

| **Location** | **Date**  **(Sample ID)** | **Time** | **Type** | **Compound**  (μg/m^3^) | **TICs**  (μg/m^3^) | **Symptoms** |
| --- | --- | --- | --- | --- | --- | --- |
| 41.1755854N 80.810184W | 10/30/13  (OH-4842) | 6:30 p.m. | Grab | Methane  (3 ppmV) |  |  |
|  | 10/29/13  (OH-4818) | 3:00 p.m. | Grab | n-Hexane (13)  n-Heptane (21)  Toluene (11)  n-Octane (27)  m,p-Xylenes (11)  n-Nonane (40)  1,2,4-Trimethylbenzene (6.8)  Methane  (2.2 ppmV) | Methylcyclohexane (22)  4-Methyloctane (22)  Decane (21)  Undecane (20) | **Smell**: Diesel fumes, tar, petroleum, chemical smell, a sweet chemical smell  **Feel**: Headache |
| 41.1755854N  80.810184W | 10/29/13  (OH-4822) | 2:00 p.m. | Grab | Methane  (2 ppmV) |  |  |
| 40.545118N 81.010255W | 10/26/13  (OH-4866) | 7:30 p.m. | Grab | Toluene (19)  Methane  (2.8 ppmV) | 2,4-Dimethylheptane (25) |  |
| 40.7515269N 81.9173190W | 10/31/13  (OH-4881-005) | 6:47 p.m. | Passive |  |  |  |
| 40.7336620N 80.9353187W | 10/31/13  (OH-4881-004) | 6:40 p.m. | Passive |  |  |  |
| 40.6833190N  80.9667705W | 10/31/13  (OH-4881-003) | 6:26 p.m. | Passive |  |  |  |
| 40.5527906N  81.0116763W | 10/31/13  (OH-4881-002) | 6:02 p.m. | Passive |  |  |  |
| 40.5607067N  81.0119634W | 10/31/13  (OH-4881-001) | 5:50 p.m. | Passive |  |  |  |

**Table S4.** Pennsylvania Grab and Passive Sample Results and Symptoms by Location.

| **Location** | **Date**  **(Sample ID)** | **Time** | **Type** | **Compound**  (μg/m^3^) | **TICs**  (μg/m^3^) | **Symptoms** |
| --- | --- | --- | --- | --- | --- | --- |
| 40.325570N  80.296268W | 9/14/13  (PA-4136) | 8:57 p.m. | Grab | n-Hexane (330)  Benzene (5.7)  Cyclohexane (25)  n-Heptane (46)  Toluene (8.2)  n-Octane (5.4)  Methane  (330 ppmV) | Propane (12,000)  Isobutane (7,700)  n-Butane (11,000)  Neopentane (120)  Isopentane (3,600)  n-Pentane (4,700)  2,2-Dimethylbutane (54)  Cyclopentane (34)  2,3-Dimethylbutane (78)  2-Methylpentane (570)  3-Methylpentane (280)  Methylcyclopentane (65)  2-Methylhexane (42)  3-Methylhexane (42)  Methylcyclohexane (35) |  |
| 41.71783N  75.87332W | 9/9/13  (PA-4082) | 6:47 p.m. | Grab | Toluene (6.7)  Chlorobenzene (8.3)  Methane (17 ppmV) |  | **Feel**: Sore throat |
| 41.71783N  75.87332W | 8/13/13  (PA-3570) | 12:02 p.m. | Grab |  |  | **Smell**: Heavy oil odor, sweet smell, burnt plastic odor  **Feel**: Sore throat, light-headed |
| 41.71783N  75.87332W | 8/13/13  (PA-3597) | 9:00 p.m. | Grab |  |  | **Smell**: Burnt oil odor  **Feel**: Light-headed, sore throat |
| 41.71745N  75.87318W | 9/20/13  (PA-4259-001) | 12:42 a.m. – 6:38 p.m. | Passive |  |  | **Feel**: Sore throat |
| 41.71783N  75.87332W | 9/20/13  (PA-4259-002) | 12:32 p.m. – 6:35 p.m. | Passive | Formaldehyde (61) |  |  |
| 41.71028N  75.91504W | 9/20/13  (PA-4259-003) | 1:36 p.m. – 6:21 p.m. | Passive | Formaldehyde (59) |  | **Feel**: Dry mouth |
| 41.71766N  75.94932W | 9/20/13  (PA-4259-006) | 1:30 p.m. – 6:15 p.m. | Passive |  |  |  |
| 41.79777N  75.82587W | 9/20/13  (PA-4259-004) | 10:40 a.m. – 5:36 p.m. | Passive | Formaldehyde (32) |  |  |
| 41.79777N  75.82587W | 9/20/13  (PA-4259-005) | 10:48 a.m. – 5:30 p.m. | Passive | Formaldehyde (34) |  |  |
| 41.71745N  75.87318W | 9/8/13  (PA-4083-001) | 3:06 p.m. – 5:30 p.m. | Passive |  |  |  |
| 41.79777N  75.82587W | 9/7/13  (PA-4083-004) | 3:50 p.m. – 3:25 p.m. | Passive | Formaldehyde (7.6) |  |  |
| 41.71783N  75.87332W | 9/7/13  (PA-4083-002) | 3:31 p.m. – 2:56 a.m. | Passive |  |  |  |
| 41.71028N  75.91504W | 9/7/13  (PA-4083-003) | 2:36 p.m. – 2:45 p.m. | Passive | Formaldehyde (8.3) |  |  |

**Table S5**. Wyoming Grab and Passive Sample Results and Symptoms by Location.

| **Location** | **Date**  **(Sample ID)** | **Time** | **Type** | **Compound**  (μg/m^3^) | **TICs**  (μg/m^3^) | **Symptoms** |
| --- | --- | --- | --- | --- | --- | --- |
| 43.1453928N  108.3735072W | 11/7/12  (WY-4586) | 1:05 p.m. | Grab | Hydrogen Sulfide (590)  n-Hexane (22,000)  Benzene (2,200)  Cyclohexane (22,000)  n-Heptane (13,000)  Toluene (1,400)  n-Octane (3,100)  Ethylbenzene (1,200)  Xylenes (4,100)  n-Nonane (1,300)  1,2,4-Trimethylbenzene (740) | Propane (330,000)  Isobutane (430,000)  n-Butane (200,000)  Neopentane (21,000)  Isopentane (230,000)  n-Pentane (82,000)  2,2-Dimethylbutane (41,000)  2,3-Dimethylbutane (67,000)  2-Methylpentane (73,000)  3-Methylpentane (46,000)  Methylcyclopentane (29,000)  2-Methylhexane (15,000)  2,3-Dimethylpentane (12,000)  3-Methylhexane (15,000)  Methylcyclohexane (40,000) | **See**: Separators, produced water tanks, pneumatic pumps, tanks, wellhead, meter sheds  **Smell**: Sickly sweet petroleum  **Feel**: Dizzy, chest tightness, nausea, headache, stiff neck, runny nose, throat irritation, acid metallic taste  **Hear**: Gas hissing, clicking of pneumatic pump, spitting and whooshing noise, rattling noise |
| 44.5942144N  108.5327261W | 10/31/13 (WY-4864) | 3:04 p.m. | Grab | Hydrogen Sulfide (210)  Methane (2.4 ppmV) | 2-Methylpentane (66)  2,4-Dimethylheptane (86)  4-Methyloctane (27)  C12H26 Branched Alkane (24-110)  Unidentified Compound (120)  Unidentified Compound (52)  Unidentified Compound (55) | **See**: Small dam, discharge canal, residue on banks of stream, discolored water  **Smell**: Rotten eggs, permanent solution, then nothing  **Feel**: Dizzy, shortness of breath, runny nose, clogged sinuses, burning eyes, heavy feeling in chest |

| 44.5937814N  108.5337174W | 10/31/13 (WY-4865) | 2:38 p.m. | Grab | Hydrogen sulfide (1,200)  Methyl Mercaptan (12)  n-Hexane (6.5)  Methane (2.4 ppmV) | n-Pentane (27)  tert-Butanol (61)  2,3-Dimethylbutane (45)  2-Methylpentane (230)  C6H12 Compound (26)  Hexamethylcyclotrisiloxane (22)  2,4-Dimethylheptane (180)  2,4-Dimethyl-1-heptene (44)  4-Methyloctane (44)  C12H26 Branched Alkane (26-120)  Unidentified Compound (110)  Unidentified Compound (47)  Unidentified Compound (56) | **See**: Discharge coming out of pipes, discolored with white residue on banks of stream, discolored water  **Smell**: Rotten eggs, permanent solution  **Feel**: Headache, burning eyes, acid taste, runny nose |
| --- | --- | --- | --- | --- | --- | --- |
| 43.156808N  108.3759006W | 10/31/13 (WY-4862) | 11:00 a.m. | Grab | Ethanol (62)  n-Hexane (30)  Cyclohexane (34)  n-Heptane (24)  Toluene (12)  n-Octane (10)  n-Nonane (6.4)  Methane (36 ppmV) | Propane (240)  Isobutane (300)  n-Butane (170)  Isopentane (190)  n-Pentane (100)  2,3-Dimethylbutane (38)  2-Methylpentane (120)  3-Methylpentane (36)  Methylcyclopentane (56)  Methylcyclohexane (98)  2,4-Dimethylheptane (52)  C12H26 Branched Alkane (64)  Unidentified Compound (73)  Unidentified Compound (34)  Unidentified Compound (38) | **See**: Separator, production tank, wellhead on location  **Smell**: sickeningly sweet petroleum smell, metallic taste in mouth |

| 43.145401N  108.373498W | 10/31/13 (WY-4861) | 10:40 a.m. | Grab | Ethanol (55)  n-Hexane (2,500)  Benzene (230)  Cyclohexane (3,500)  n-Heptane (980)  Toluene (110)  n-Octane (320)  Ethylbenzene (52)  m,p-Xylenes (290)  o-Xylene (27)  n-Nonane (110)  Cumene (11)  n-Propylbenzene (12)  4-Ethyltoluene (12)  1,3,5-Trimethylbenzene (29)  1,2,4-Trimethylbenzene (47)  Methane (5,900 ppmV) | Propane (10,000)  Isobutane (14,000)  n-Butane (7,500)  Isopentane (13,000)  n-Pentane (7,200)  2,2-Dimethylbutane (1,800)  Cyclopentane (1,200)  2,3-Dimethylbutane (2,700)  2-Methylpentane (7,300)  3-Methylpentane (3,800)  Methylcyclopentane (5,000)  2-Methylhexane + 2,3-Dimethylpentane (1,800)  3-Methylhexane (1,200)  Methylcyclohexane (5,400)  Dimethylcyclohexane (1,300) | **See**: Separators, produced fluid tanks **Smell**: Sickeningly sweet petroleum smell  **Feel**: Dizzy, nausea, headache, metallic taste, nose/throat irritation, aches in joints  **Hear**: Hissing sounds and sounds like pressurized gas is leaking |
| --- | --- | --- | --- | --- | --- | --- |
| 44.5557745N  109.51115W | 7/30/13  (WY-3321) | 9:30 a.m. | Grab | n-Hexane (770)  Benzene (35)  Cyclohexane (420)  n-Heptane (190)  Toluene (89)  n-Octane (65)  m,p-Xylenes (22)  o-Xylene (6.2)  n-Nonane (17) | Propane (1,700)  Isobutane (3,900)  n-Butane (4,900)  Isopentane (3,800)  n-Pentane (3,300)  2,2-Dimethylbutane (110)  Cyclopentane (310)  2-Methylpentane (1,200)  3-Methylpentane (620)  Methylcyclopentane (790)  2-Methylhexane (100)  3-Methylhexane (110)  Methylcyclohexane (370)  C12H26 Branched Alkane (140)  C13H28 Branched Alkane (120) | **See**: Building with open door, pipes, equipment, puddle of leaked oil  **Smell**: Hydrocarbons, chlorine  **Feel**: Heat emitting from machinery, headache (light), runny nose, burning eyes  **Hear**: Drone of the engine, fan turning, rushing air |

| 44.5835133N  109.1515476W | 10/29/12  (WY-4478) | 12:01 p.m. | Grab | Hydrogen Sulfide (91)  Ethyl Mercaptan (58)  Isopropyl Mercaptan (540)  tert-Butyl Mercaptan (25)  n-Propyl Mercaptan (78)  Thiophene (220)  Isobutyl Mercaptan (84)  Diethyl Sulfide (41)  Tetrahydrothiophene (22)  2-Ethylthiophene (28)  n-Hexane (1,200,000)  Benzene (110,000)  Cyclohexane (690,000)  n-Heptane (480,000)  Toluene (270,000)  n-Octane (180,000)  Ethylbenzene (17,000)  m,p-Xylenes (110,000)  o-Xylene (25,000)  n-Nonane (35,000)  1,2,4-Trimethylbenzene (6,400)  d-Limonene (5,600) | Propane (1,900,000)  Isobutane (4,000,000)  n-Butane (4,100,000)  Isopentane (3,700,000)  n-Pentane (3,100,000)  2,2-Dimethylbutane (230,000)  Cyclopentane (320,000)  2,3-Dimethylbutane (400,000)  2-Methylpentane (1,700,000)  3-Methylpentane (990,000)  Methylcyclopentane (1,200,000)  2-Methylhexane (320,000)  3-Methylhexane (300,000)  Dimethylcyclopentane Isomer (160,000)  Methylcyclohexane (1,100,000) | **See**: Vapor flow/venting tubes, brown liquid drip, oily substance on ground and buildup on tubes  **Feel**: Headache, tightness in neck, throat soreness, runny nose, dizziness, burning eyes, metallic taste  **Hear**: Hissing, clicking and whooshing |
| --- | --- | --- | --- | --- | --- | --- |
| 43.157632N  108.3659577W | 5/15/13  (WY-2069) | 8:40 p.m. | Grab | Hydrogen Sulfide (30)  Ethanol (61)  n-Hexane (200)  Cyclohexane (120)  n-Heptane (71)  Toluene (12)  n-Octane (22)  n-Nonane (5.6) | Propane (2,300)  Isobutane (2,700)  n-Butane (1,600)  Neopentane (52)  Isopentane (1,400)  n-Pentane (800)  2,2-Dimethylbutane (75)  2,3-Dimethylbutane (110)  2-Methylpentane (410)  3-Methylpentane (200)  Methylcyclopentane (230)  2-Methylhexane (58)  Methylcyclohexane (250)  C12H26 Branched Alkane (82)  C15H32 Branched Alkane (68) | **See**: Workover rig and fracking tanks  **Smell**: Sickly sweet hydrocarbon smell  **Feel**: Headache, nausea, confusion, shakes, vomit taste in back of mouth  **Hear**: Hissing and chirping sounds |

| 43.156808N  108.3759006W | 3/19/13  (WY-1103) | 12:45 p.m. | Grab | Propene (6.9)  Ethanol (52)  n-Hexane (350)  Benzene (31)  Cyclohexane (310)  n-Heptane (190)  Toluene (71)  n-Octane (71)  Ethylbenzene (9.5)  m,p-Xylenes (53)  o-Xylene (11)  n-Nonane (32)  1,3,5-Trimethylbenzene (5.9)  1,2,4-Trimethylbenzene (8.5) | Propane (2,000)  Isobutane (2,600)  n-Butane (1,700)  Neopentane (76)  Isopentane (1,700)  n-Pentane (1,000)  2,2-Dimethylbutane (170)  Cyclopentane (92)  2,3-Dimethylbutane (270)  2-Methylpentane (780)  3-Methylpentane (380)  Methylcyclopentane (470)  2-Methylhexane (140)  3-Methylhexane (140)  Methylcyclohexane (730) | **See**: Gas spewing from top of production tank  **Smell**: Sickly sweet petroleum  **Feel**: Headache, nausea, throat irritation, felt uncomfortable, metallic taste, chest tightness, mental confusion |
| --- | --- | --- | --- | --- | --- | --- |
| 44.552636N  108.39618W | 1/16/13  (WY-0187) | 11:00 a.m. | Grab | Hydrogen Sulfide (66,000)  Methyl Mercaptan (47)  n-Hexane (22)  Benzene (23)  Cyclohexane (39)  n-Heptane (8.2)  Toluene (100)  Ethylbenzene (28)  m,p-Xylenes (95)  o-Xylene (46)  4-Ethyltoluene (5.2)  1,3,5-Trimethylbenzene (6.3)  1,2,4-Trimethylbenzene (17) | Carbonyl Sulfide (64)  Propane (280)  Sulfur Dioxide (>860)  Isobutane (240)  n-Butane (320)  Isopentane (260)  n-Pentane (90)  Cyclopentane (68)  2-Methylpentane (53)  3-Methylpentane (57)  Methylcyclopentane (68)  Methylcyclohexane (27)  C12H26 Branched Alkane (28)  C15H32 Branched Alkane (37)  C16H34 Branched Alkane (30) | **See**: Riparian area, green scummy water and green ice  **Smell**: Very heavy H_2_S density  **Feel**: Very light-headed coming out  **Hear**: Clanging, screeching well, humming noise |

| 44.56411N  108.3914227W | 1/16/13  (WY-0184) | 10:00 a.m. | Grab | Hydrogen Sulfide (240)  Toluene (11) | Sulfur Dioxide (>29)  C12H26 Branched Alkane (21)  C15H32 Branched Alkane (39)  C15H32 Branched Alkane (30)  C16H34 Branched Alkane (36) | **See**: Discharge water from wells flows into irrigation water drains and watershed where goats drink and pasture, green slime, paraffin, and film in water  **Smell**: Heavy H_2_S,  toxic smell  **Feel**: Sick  **Hear**: Thumping, squealing of wells |
| --- | --- | --- | --- | --- | --- | --- |
| 44.5654431N  109.1018091W | 1/10/13  (WY-0129) | 2:52 p.m. | Grab | Ethanol (55)  n-Hexane (210)  Benzene (100)  Cyclohexane (170)  n-Heptane (28)  Toluene (48) | Propane (930)  Isobutane (740)  n-Butane (1,200)  Isopentane (820)  n-Pentane (670)  2,2-Dimethylbutane (38)  C5H10 Compound (88)  2,3-Dimethylbutane (61)  2-Methylpentane (300)  3-Methylpentane (180)  Methylcyclopentane (280)  2-Methylhexane (22)  3-Methylhexane (21)  Methylcyclohexane (96) | **See**: Flare burning and smokestacks  **Smell**: Gasoline/ petroleum; like a stove with unlit pilot light.  **Feel**: Burning nose, headache, light-headed  **Hear**: Humming and roaring |
| 44.581201N  109.143530W | 1/9/13  (WY-0105) | 1:51 p.m. | Grab | n-Hexane (11)  Toluene (7.7) | Propane (190)  Isobutane (80)  n-Butane (100)  Isopentane (69)  n-Pentane (46)  C13H28 Branched Alkane (24) | **See**: Tubes coming out the side of source building, stains on ground, brown crusty residue on the tubes  **Smell**: Gasoline/  hydrocarbon smell  **Feel**: Light-headed, headache, nose burned, eyes watered  **Hear**: Whooshing and clicking at source |

| 44.552595N  108.39751W | 1/9/13  (WY-0106) | 11:45 p.m. | Grab | Hydrogen Sulfide (5,600)  Ethanol (55)  Ethyl Acetate (13)  Toluene (22)  o-Xylene (5.1) | Sulfur Dioxide (>220)  C12H26 Branched Alkane (33)  C13H28 Branched Alkane (41) | **See**: Drainage ditch supposedly clean final discharge where livestock and wildlife would drink is cloudy, nasty, toxic discharge water and very oily if a stick is inserted (or animals walked through) two feet down in the soil. No oil well pad liners; water from pad is also leaching and freezing = ice flow into our pastures. Oil spill from pipe (taking oil to factory)  **Smell**: Very strong H_2_S gas odor, raw oil near wellhead itself, very strong as we walk back through well pad  **Feel**: Cognitive problems such as cloudy/foggy thinking, dizziness, very heavy H_2_S fume smell for short time while taking sample makes us feel woozy, dizzy, cloudy, and foggy. I wanted to come home and sleep. My chest also feels tight  **Hear**: Presence of oil well nearby – droning motor, banging sounds endless, squealing of machine parts as the pumper pumps its oil. Very noisy, constant. Can hear these at our residence |
| --- | --- | --- | --- | --- | --- | --- |

| 44.5525551N  108.398177W | 10/31/12 (WY-4496) | 12:19 p.m. | Grab | Hydrogen Sulfide (6,100)  Ethyl Acetate (11)  Benzene (9.2)  Toluene (23)  m,p-Xylenes (12)  o-Xylene (6.8) | Sulfur Dioxide (>220)  Isobutane (36)  n-Butane (43) | **See**: Oil sheen, scum on ponds, irrigation water filthy, inadequate land farming operations to clean soil  **Smell**: Rotten egg, dirty/oily, noxious fumes  **Feel**: Very sick, light-headed, heavy chest |
| --- | --- | --- | --- | --- | --- | --- |
| 44.5557745N  109.51115W | 10/30/13  (WY-4883-005) | 9:07 a.m.-5:05 p.m. | Passive | Formaldehyde (46) |  |  |

**Table S6.** Grab Samples Collected on Pollution Patrol in Fremont and Park Counties, Wyoming.

| **Date/**  **Time** | **Nearest Infrastructure** | **Distance (feet)** | **Production Stage** | | | | **Importance of Sample Location** | **Anomalies**  **Reported** |
| --- | --- | --- | --- | --- | --- | --- | --- | --- |
|  |  |  | **Production** | | **Development** | |  |  |
|  |  |  | **Oil** | **Gas** | **Oil** | **Gas** |  |  |
| 10/31/13  3:04 p.m. | Christmas trees^a^  Compressor stations  Dehydrator units  Discharge canal  Production tanks  Pump jacks | 20 | X | X |  |  | Siphon/dam in discharge canal  Along county road | Discolored water-discharge canal  Residue on canal banks  Strong H_2_S odor |
| 10/31/13  2:38 p.m. | Christmas trees^a^  Compressor stations  Dehydrator units  Discharge canal  Production tanks  Pump jacks | 5 | X | X |  |  | Along county road | Foul-smelling/discolored discharge water  White residue on canal banks  Strong H_2_S odor |
| 10/31/13  11:00 a.m. | Christmas tree^a^  Produced water condensate tanks  Separation equipment  Separator  Well | 50 |  | X |  |  | 3/4 mi. SW of home  Known for heavy emissions  Cows fed 2x/d nearby | Oil stains and slicks on pad  Inadequate fencing  Strong petroleum odors |
| 10/31/13  10:40 a.m. | Christmas trees^a^  Produced water condensate tanks  Separation equipment  Separators (3)  Wells (5) plumbed to pad | 15 |  | X |  |  | Very close to a home  In cow pasture | Oil stains and slicks on pad  Inadequate fencing  Strong petroleum odors  Sounds like gas is leaking |
| 7/30/13  9:30 a.m. | Compressor building  Discharge ponds  Pipelines  Pump jack  Separation equipment  Storage tanks  Ventilation fans | 10 |  |  | X | X | Near church, residences  Along county road | Open doors  Spills, leaks  Damaged fences  Garbage  Heat from machinery  Hydrocarbon odors |

| 5/15/13  8:40 a.m. | Christmas trees^a^ (2)  Fracking fluid tanks  Produced water condensate tanks  Wells (3-4) plumbed to pad  Workover rig | 350-400 |  | X |  |  | 600 feet from home  1/8 mi. from home  1/4 mi. from home | Hissing, dripping sounds  Sweet hydrocarbon odors |
| --- | --- | --- | --- | --- | --- | --- | --- | --- |
| 3/19/13  12:45 p.m. | Christmas tree^a^  Produced water condensate tanks  Separation equipment | 40 |  | X |  |  | 3/4 mi. SW of home  Known for heavy emissions  Cows fed 2x/d nearby | Strong hydrocarbon/sweet chemical odors  Gas spewing from top of production tank |
| 1/16/13  11:00 a.m. | Oil tanks  Produced water impoundments (3)  Pump jack  Well pad | 240 | X |  |  |  | Within goat/cow pastures  Spills, leaks on private land  Oil bogs from pipelines  Produced water degradation  Livestock disease, deaths | Used oil socks  Hydrocarbon spills  Inadequate fencing  Scum, oily sheen on impoundments  Tanks/infrastructure in poor condition  Rusted fences and metal  Strong H_2_S odor  Garbage |
| 1/16/13  10:00 a.m. | Oil tanks  Produced water impoundments (3)  Pump jack  Well pad | 1050 | X |  |  |  | Within goat/cow pastures  Spills, leaks on private land  Oil bogs from pipelines  Produced water degradation  Livestock disease, deaths | Used oil socks  Hydrocarbon spills  Inadequate fencing  Scum, oily sheen on impoundments  Tanks/infrastructure in poor condition  Rusted fences and metal  Strong H_2_S odor  Garbage |

| 1/10/13  2:52 p.m. | Compressor station  Condensate storage tanks  Flare stacks  Loading yard for condensate  Pipeline junctures  Separation equipment  Storage facility for pipes, equipment | 20 |  | X |  |  | In middle of rural subdivision  4 homes w/in 1/2 mi.  10 homes w/in 1 mi.  20 homes w/in 2 mi. | Inadequate holding tanks  Homemade vents  Damaged buildings  Loud compressors  Leaks, spills  Air emissions  Condensate drips, pooling  Strong hydrocarbon odors |
| --- | --- | --- | --- | --- | --- | --- | --- | --- |
| 1/9/13  1:51 p.m. | Christmas trees^a^ (4)  Fracking fluid tanks  Frost-free water pump  Pneumatic pumps  Produced water tanks  Pump jack (large)  Separator sheds  Well housing for water well | 60 |  | X |  |  | Pad in middle of subdivision  15 homes w/in 1/2 mi.  20 homes w/in 1 mi.  50 homes w/in 2 mi. | Leaks, spills  Open and unlocked separator shed doors |
| 1/9/13  11:45 a.m. | Oil tanks  Produced water impoundments (3)  Pump jack  Well pad | 90 | X |  |  |  | Within goat/cow pastures  Spills, leaks on private land  Oil bogs from pipelines  Produced water degradation  Livestock disease, deaths | Used oil socks  Hydrocarbon spills  Inadequate fencing  Scum, oily sheen on impoundments  Tanks/infrastructure in poor condition  Rusted fences and metal  Strong H_2_S odor  Garbage |
| 11/7/12  1:05 p.m. | Christmas trees^a^ (2)  Meter sheds  Produced water tanks  Wells (5) plumbed to pad | 1 |  | X |  |  | SW of home  Known for heavy emissions  Cows fed 2x/d nearby | Leaks, spills  Clicking pneumatic pumps  Rattling equipment  Strong hydrocarbon/sweet chemical odors  Hissing sound |
| 10/31/12  12:19 p.m. | Oil tanks  Produced water impoundments (3)  Pump jack  Well pad | 10 | X |  |  |  | Within goat/cow pastures  Spills, leaks on private land  Oil bogs from pipelines  Produced water degradation  Livestock disease, deaths | Used oil socks  Hydrocarbon spills  Inadequate fencing  Scum, oily sheen on impoundments  Tanks/infrastructure in poor condition  Rusted fences and metal  Strong H_2_S odor  Garbage |
| 10/29/12  12:01 p.m. | Christmas trees^a^ (2)  Pipeline junction  Produced water condensate tanks  Separator shed | 1 |  | X |  |  | Pad in middle of subdivision  5 homes w/in 1/4 mi.  10 homes w/in 3/4 mi.  Gas well blowout (2006) | Clicking, whooshing, hissing sounds  Vapors from emissions point  Liquid dripping from tubes/separator shed  Staining on ground |

X indicates affirmation of stage of development of oil or gas

^a^ = an assembly of valves and fittings on top of a well casing
